# Analysis of the Characteristics of CH_4_ Emissions in China’s Coal Mining Industry and Research on Emission Reduction Measures

**DOI:** 10.3390/ijerph19127408

**Published:** 2022-06-16

**Authors:** Anyu Zhu, Qifei Wang, Dongqiao Liu, Yihan Zhao

**Affiliations:** 1 School of Emergency Management and Safety Engineering, China University of Mining and Technology, Beijing 100083, China; zhuanyu520@126.com; 2 School of Mechanical-Electronic and Vehicle Engineering, Beijing University of Civil Engineering and Architecture, Beijing 102616, China; zhaoyihan142@163.com; 3 State Key Laboratory for Geomechanics and Deep Underground Engineering, China University of Mining and Technology, Beijing 100083, China; liudongqiao@yeah.net

**Keywords:** methane emissions, coal mining, CH_4_ emission factor, China

## Abstract

CH_4_ is the second-largest greenhouse gas and has a significant impact on global warming. China has the largest amount of anthropogenic coal mine methane (CMM) emissions in the world, with coal mining emissions (or gas emissions) accounting for 90% of total energy industry emissions. The results of CH_4_ emission inventories from previous studies vary widely, with differences in the spatial and temporal dimensions of gas emission factors of belowground mining being the main points of disagreement. Affected by the policies of “eliminating backward production capacity” and “transferring energy base to the northwest”, China’s coal production layout has changed greatly in the past ten years, but the closely related CH_4_ emission factors have not been dynamically adjusted. This paper investigated 23 major coal producing provinces in China, obtained CH_4_ emission data from coal mining, calculated CH_4_ emission factors in line with current production conditions, and studied the reduction measures of coal mine gas emission. According to the CH_4_ emission data of China’s coal mines in 2018, 15.8 Tg of methane is released per year in the coal mining industry in China, and 11.8 Tg after deducting recycling. Shanxi Province’s CH_4_ emissions are much higher than those of other provinces, accounting for 35.5% of the country’s total emissions. The weighted CH_4_ emission factor of coal mining in China is 6.77 m^3^/t, of which Chongqing is the highest at approximately 60.9 m^3^/t. Compared with the predicted value of the IPCC, the growth trend of CCM has slowed significantly, and the CH_4_ utilization rate has gradually increased. This change may be aided by China’s coal industry’s policy to resolve excess capacity by closing many high-gas and gas outburst coal mines. In addition, the improvement of coal mine gas extraction and utilization technology has also produced a relatively significant effect. This paper determines the distribution of methane emissions and emission sources in China’s coal mining industry, which is useful in formulating CCM emission reduction targets and adopting more efficient measures.

## 1. Introduction

After CO_2_, CH_4_ is the second-largest greenhouse gas in the world, and its 20-year global warming potential (GWP) is 84 times that of CO_2_ [1]. Although the composition of Coal Bed Methane (CBM) varies widely around the world, the main components of CBM are CH_4_ (more than 80%), other hydrocarbons and small amounts of CO_2_ and N_2_. In addition, methane is also a key non-CO_2_ greenhouse gas, which is also the current concern of China. The Glasgow Joint Declaration between China and the United States on Strengthening Climate Action in the 2020s mentioned that: China and the United States are particularly aware of the significant impact of CH_4_ emissions on global warming and believe that it is necessary to take further measures to reduce CH_4_ emissions in the 1920s. The sixth assessment report (AR6) issued by the Intergovernmental Panel on Climate Change (IPCC), the third working group report “Climate Change 2022: Mitigating Climate Change” pointed out that to achieve the goal of limiting global warming to 1.5 °C, it is necessary to pay attention to the reduction of CH_4_ [1].

A study by McKinsey and Company reports Safe to work shows that the entire mining industry emits between 1.9 and 5.1 gigatons of carbon dioxide annually. At the same time, uncontrolled coal-bed methane provides from 1.5 to 4.6 gigatons of emissions. Methane has significant negative effects on human health and ecosystems: 90% of methane in the atmosphere will be oxidized by hydroxyl radicals, which will promote the formation of atmospheric ozone, and the ozone in the troposphere will endanger human health. Moreover, the increase in ozone concentration may induce luminous chemical smog, which has a more adverse impact on human health. On 11 August 2021, the United Nations Intergovernmental Panel on Climate Change (IPCC) released the Sixth Assessment Report Working Group I report, further emphasizing the importance of methane reduction. A discussion about the role of methane emission reduction is expounded in slowing global warming and improving air quality, and it is pointed out that if methane emission is not controlled, mankind will not be able to achieve the goal of controlling the temperature rise proposed in the Paris Agreement.

CH_4_ emission sources mainly include natural emission sources and anthropogenic emission sources [2]. Natural emissions consist mostly of wetlands, vegetation, oceans, and methane hydrate, while anthropogenic emissions mainly include energy, agricultural activities, waste treatment, and constructed wetlands. In energy activities, CH_4_ is emitted from coal mining (mainly underground mining), coal processing, and transportation [3]. CH_4_ is emitted the most during mining because the CH_4_ stored in the coal seam and surrounding rock formations escaping when the coal seam is mined [3]. As the largest coal producing and consuming country, China has a vast amount of CH_4_ emissions caused by coal mining [2]. According to the Greenhouse Gas Emission Inventory released by the Ministry of Ecology and Environment of China, China’s total CH_4_ emissions from coal mining activities in 2018 were 20.045 Tg, accounting for 38% of China’s total anthropogenic methane emissions, which is far higher than the global level [2]. Therefore, controlling CH_4_ emissions from coal mining is of great significance in the realization of China’s carbon peaking, carbon neutrality, and global greenhouse cutting goals.

An important prerequisite for controlling CH_4_ emissions from coal mining is to accurately assess the CH_4_ emissions from coal mining in China and their spatial distribution characteristics [4]. Some scholars and research institutions have evaluated the methane emissions from coal mining in China. Yuan et al. calculated the emissions of pollutants including CH_4_ during the extraction of fossil fuels in China in 2002 [5]. Zhang et al. provided CH_4_ emission inventories from 1980 to 2007, 2008, and 2010 [6,7,8]. Peng et al. reported China’s anthropogenic CH_4_ emissions inventory from 1980 to 2010 and drew the provincial emission maps [9]. Gong et al. provided a monthly inventory of CH_4_ emissions from natural and anthropogenic sources in China in 2015 [10]. Schwietzke et al. studied the CH_4_ emissions inventories from coal mining in countries around the world, including China, from 1980 to 2011 [11]. Höglund et al. estimated CH_4_ emissions for the five-year interval 2005 to 2030 [12]. However, there are large differences in the evaluation results between the different studies mentioned above, and the different rate of the evaluation in some years is as high as 75%. This is mainly because there are differences in the evaluation methods and emission factors selected during different evaluation processes in the above studies [2]. The authors mainly used three methods to calculate CH_4_ emissions from coal mining. Method 1 uses the global average methane emission factor, method 2 uses the methane emission factor of a country or a specific region, while method 3 calculates CH_4_ emissions from coal mining based on measured data of every coal mine [13]. In terms of the emission factors, CH_4_ emissions from underground coal mining in China are mainly estimated based on the default national emission inventory and the emission factors recommended by the IPCC. These data parameters have large errors in calculating the overall emissions profile. In addition, most of the methane emission data referenced in the existing research were published before 2015, while approximately 5500 coal mines were closed nationwide during the “13th Five-Year Plan” period [14], most of which were small and medium-sized gassy coal mines. Therefore, the above data cannot objectively and truly reflect the current CH_4_ emissions from coal mining in China.

Based on existing research, this paper selected a method for calculating coal mine CH_4_ emissions that is in line with China’s national conditions. Data related to the calculation of CH_4_ emissions from 4818 coal mines in 23 major coal-producing provinces in China were collected and then screened. According to the measured data of coal mines in each province, the average CH_4_ emission factor of coal mining in the province is calculated. Finally, the total CH_4_ emissions from coal mining in China in 2018 and the spatial emission characteristics of coal mine methane are evaluated. Relevant research can provide data support and decision-making references for the formulation of CH_4_ emission reduction policies in the coal industry in the context of carbon neutrality and carbon peaking proposed by the Chinese government.

## 2. Materials and Methods

### 2.1. Method for Estimating CH_4_ Emissions

Coal mining includes underground mining and open-pit mining, with the former generally having higher CH_4_ emissions. The United Nations Intergovernmental Panel on Climate Change (IPCC) provides three methods for calculating CH_4_ emissions from coal mining [13]. Method 1 is a default method, using the global average CH_4_ emission factor, which can be used to roughly estimate CH_4_ emissions from coal mining. Method 2 considers the characteristics of different regions and selects the CH_4_ emission factors of countries or specific regions, and is more accurate during calculation than method 1. Method 3 calculates the CH_4_ emissions caused by coal mining based on the measured data, also known as the direct measurement method, and the evaluation results of this method are the most accurate. According to the above analysis, it is necessary to determine the CH_4_ emission factors in coal mining (as shown in Table 1) before calculating the CH_4_ emissions using method 1 or method 2, and then multiply the corresponding coal output to obtain the total CH_4_ emission. These two methods are simpler and more convenient to calculate, but the results are less accurate. Method 3 does not need to determine the CH_4_ emission factor, but directly sums the measured CH_4_ emission data of each mine to obtain the total CH_4_ emission, which is more accurate, but it is very difficult to operate in the implementation process because we must obtain the field measured data of all mines. Usually, method 3 is only suitable for countries with few coal mines.

### 2.2. Method for Estimating CH_4_ Emissions from Coal Mining in China

China is the largest coal producer in the world. Coal mines are widely distributed, with an annual output of more than 5 million tons in more than 20 provinces. However, the spatial distribution of coal production and methane emissions caused by coal mining is quite uneven. The gas content in coal in northwest provinces such as Xinjiang and Inner Mongolia is very tiny. In contrast, most coal mines in southwest provinces such as Chongqing and Sichuan are high-gas mines, and in some of them, the gas content can reach tens of cubic meters per ton. Therefore, the international general CH_4_ emission factor recommended by the IPCC does not reflect the actual situation in China. However, the direct measurement method needs to obtain the CH_4_ emission data of all coal mines in the country, which consumes considerable material resources and time, and the implementation cost is high. Therefore, it is more scientific and reasonable to use method 2 to calculate the CH_4_ emissions caused by coal mining in China. The calculation formula is as follows:

(1) $$E=\sum_{i}P_{i}\times EF_{i}\times t-r$$

where index *i* denotes the different producing underground mining; *E* represents the CH_4_ emission (the amount of CH_4_ released into the air); *P_i_* is the amount of coal production; *EF_i_* is the CH_4_ emission factor; *t* is the gas unit conversion factor; and *r* is the methane recovered and utilized for energy production or flared.

Coal mine production is one of the main factors affecting CH_4_ emissions from coal mines. International Energy Agency (IEA) [12], China Statistical Yearbook (CSY) [5], China Energy Statistical Yearbook (CESY) [9], US Energy Information Administration (USEIA) [11], State Administration of Coal Mine Safety Supervision of China (SACMS) [15], and other institutions regularly disclose the underground and opencast coal mines of countries and regions coal mine production data. The estimation of the scholars mentioned above are calculated based on these data. There is a small gap between the data from different sources. The coal mine production data of each province in China to be used next in this paper come from the China Energy Statistical Yearbook. The CH_4_ emission factors of underground coal mining and open-pit coal mining are quite different, as shown in Table 1, the former is generally more than ten times that of the latter. Moreover, according to data released by CESC and CESY, the output of China’s open-pit coal mines accounts for only approximately 15% of the total coal mine output. In terms of total emissions, the total CH_4_ emissions from underground mining account for 95% of the total CH_4_ emissions from coal mining in the country, while open-pit mining accounts for only 5% [9]. Since the CH_4_ emissions in open-pit mining account for a very low proportion of China’s total CH_4_ emissions, this paper mainly discusses the estimation of CH_4_ emission factors in underground mining, and the relevant parameters of open-pit mining can directly refer to the values recommended by the IPCC.

For the recovered and utilized CH_4_, the values taken in the study are also different. For example, in the study of Yuan et al. [5], the utilization rate of mine gas accounted for 15% to 20%, and Huang et al. [3] used the average theoretical recovery rate of coalbed CH_4_ to be 27%. In fact, with the improvement of coal gas extraction technology, the utilization rate of mine CH_4_ is also increasing.

For the CH_4_ emissions of underground coal mines, the emission factors of different mines are also distinct due to the various conditions such as the mining depth and the gas content of each coal layer. Most studies use national or provincial CH_4_ emission factor methods to assess CH_4_ emissions, calculated by self-measurement, or emission factors obtained from previous studies. According to the methane content of mines, underground mines are divided into high- and low-gas mines in the National Research on Climate Change of China, and the average CH_4_ emission factor of high-gas mines in China is 21.83 m^3^/t. The emission factor for low-gas mines is assumed to be the coalbed CH_4_ content, and the CH_4_ emission factor for low-gas mines is 4.53 m^3^/t [16]. Zheng Shuang’s research obtained the weighted average CH_4_ emission factors of coal production in China’s major coal-producing regions based on survey data from 1994 and 2000 [17]. Considering the high proportion of high-gas and outburst mines in China, which is quite different from the other countries, most studies use the CH_4_ emission factors given by Zheng Shuang [4,8,9,14]. In addition, some experts and scholars use data from other sources to classify the CH_4_ emission factors of each province in China. Sheng also considered the differences in separate regions of China’s coal mines, and calculated the provincial average CH_4_ emission factors based on the relevant data of 10,963 coal mines given by SACMS in 2011 [15]. Zhu calculated the CH_4_ emissions factor based on the data of 787 coal mines published by SACMS in 2009, established a gray prediction model to estimate the gas emission factor from 2006 to 2010, and predicted the CH_4_ emission factors and coal mines from 2011 to 2020 [18]. 

Most of the above calculation methods are based on a small amount of measured data many years ago, but the production layout of China’s coal mines has undergone significant changes in the past 10 years, as have the associated coal mine CH_4_ emission factors and emissions. On the one hand, with increasing mining depth, some coal mines have evolved from low-gas to high-gas mines. On the other hand, the Chinese government has continued to optimize the coal mine capacity structure. For example, from 2016 to 2018, the outdated production capacity of 810 million tons (most of which are high-gas mines or outburst mines) was eliminated, and a new batch of 10 million-ton low-gas mines was put into production. Undoubtedly, the CH_4_ emission factor of each province has also changed accordingly. Therefore, it is urgent to study more accurate CH_4_ emission factors based on China’s current national conditions in combination with the current situation, and establish a unified measurement standard for CH_4_ emissions.

### 2.3. Data Source and Calculation Process

The IPCC recommends that best efforts should be made to obtain measured data when underground mining is the main source of emissions [15]. Extensive field data collection is the basis for the estimation of gas emissions from underground coal mining. In this paper, we adopt method 2 to conduct research. First, the data of some coal mines are sampled, and then the provincial CH_4_ emission factor weighted by coal mine production is obtained. Finally, the gas emissions of the whole province are estimated.

The research object of this research is the underground mining coal mines that were in production in 2018. The research covers 23 major coal-producing provinces, including Inner Mongolia, Shanxi, Shaanxi, Xinjiang, Henan, Guizhou, Shandong, Anhui, Heilongjiang, Ningxia, Hebei, Sichuan, Liaoning, Gansu, Yunnan, Jilin, Chongqing, Jiangsu, Hunan, Qinghai, Jiangxi, Guangxi, and Hubei. A total of 4818 coal mines were investigated in this research, accounting for 83% of the national production mines. The main parameters investigated include the actual production of coal mines and the absolute gas emission rate. In fact, the process of surveying many small and medium-sized coal mines is not ideal, and there are many deficiencies and irregularities in the collected data. After excluding the nonstandard or unreasonable data, this study obtained effective data from 1840 coal mines, accounting for 31.7% of the total number of coal mines in China. The combined production capacity of these coal mines is 1.808 billion tons, accounting for 45.4% of China’s total coal production capacity.

Absolute gas emission is the amount of gas emitted from coal seams and mined coal (rock) per unit time, and its unit is m^3^/min. The calculation method is the integration of the gas concentration in the return air flow multiplied by the air volume per minute. The gas concentration data can be obtained directly from the coal mining monitoring system, and the air volume can be obtained by multiplying the wind speed measured by the wind speed sensor in the monitoring system by the cross-sectional area of the roadway. The formula for calculating the annual CH_4_ emissions through absolute gas emissions is:

(2) EA=T×ea

where *EA* denotes the total gas emission or CH_4_ emission of a coal mine; *T* is the actual mining time of a coal mine, which is usually continuous mining 24 h a day throughout the year in China; and *ea* is the absolute gas emission of a coal mine.

Since it is impossible to directly obtain the CH_4_ emission data of all coal mines, it is necessary to estimate the CH_4_ emission factors of each province based on the sampling survey. The specific research and estimation methods are as follows:

(1) With the assistance of the government’s energy department, conduct research on coal mines in China’s major coal-producing provinces by email, telephone, and on-site consultation, and collect data on coal mine production and absolute gas emissions.
(2) Calculate the annual CH_4_ emissions of each mine using Formula (2).
(3) According to the data collected in each province, the cumulative gas emissions and coal production of all coal mines are calculated, and then the CH_4_ emission factor of the province’s production-weighted average is calculated.

The formula for calculating the CH_4_ emission factor is as follows:

(3) $$EFA=\frac{\sum_{1}^{m}EA_{i}}{\sum_{1}^{m}P_{i}}$$

where *EFA* is the emission factor calculated from the absolute gas emission; index *i* denotes the different producing underground mines; m is the number of producing mines in the province; *EA_i_* is the total gas emission or CH_4_ emission of the mine; *P_i_* is the output of each coal mine.

To show the calculation process of the mine CH_4_ emission factor more clearly, the whole calculation process is described in detail by selecting Qinghai Province as an example. There are 18 active coal mines in Qinghai Province, with a total output of 12.2 million tons. Through the investigation, the mining depth, coal mine output, absolute gas emission, and other data of nine coal mines were obtained. The CH_4_ emissions of these nine coal mines were calculated by Formula (2), and the CH_4_ emission factors were calculated by Formula (3). The calculation results are shown in Table 2. It can be seen from Table 2 that the production-weighted average CH_4_ emission factor of the coal mining process in Qinghai Province is 5.29 m^3^/t.

## 3. Results and Discussion

### 3.1. CH_4_ Emission Characteristics in Coal Mining

**Figure 1:**
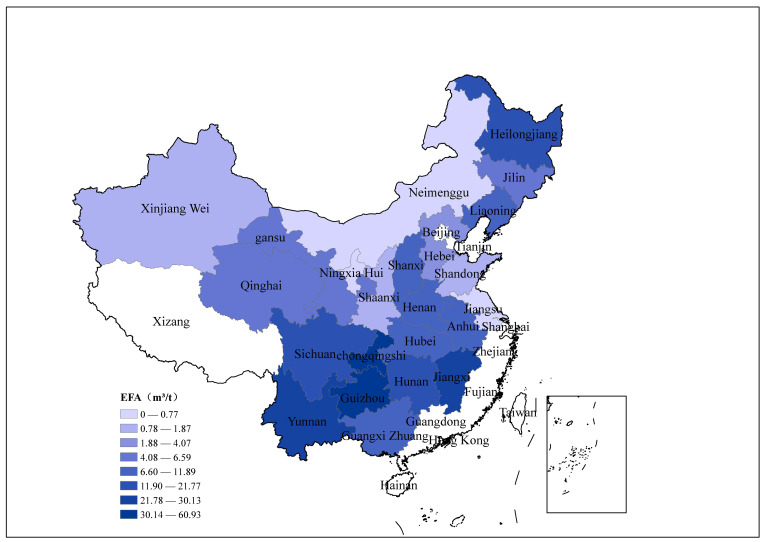
CH_4_ emission factors of the coal mining industry in various provinces.

According to the evaluation of this study, the weighted average CH_4_ emission factor of China’s coal mining industry in 2018 was 6.77 m^3^/t, which is close to the lower limit of China’s national emission factor range: 6.87–11 m^3^/t [2]. However, there are significant differences among different provinces, and the CH_4_ emission factors by province are shown in Figure 1. Figure 1 shows the CH_4_ emission factors of the provinces in the southwest region are the highest, followed by those in the northeast region. Southwest China also has the most serious gas disasters in China. Among them, the CH_4_ emission factor in Chongqing is the highest, approximately 60.9 m^3^/t. The main reason is that there are many high-gas mines and outburst mines in Chongqing, which is one of the areas with the most serious coal and gas outbursts in China [19]. According to our survey results, 95% of the coal mines in Chongqing are high-gas or gas outburst mines. In addition, the CH_4_ emission factors in Jiangsu, Inner Mongolia, Shandong, Shanxi, Ningxia, and Xinjiang are relatively low, with none exceeding 2 m^3^/t. In recent years, China’s national coal production center has continued to focus on regions with good resource endowments such as Shaanxi, Inner Mongolia, and Xinjiang, and production in these provinces has increased significantly. This is also the key reason for the low weighted emission factor of China’s national coal production.

**Figure 2:**
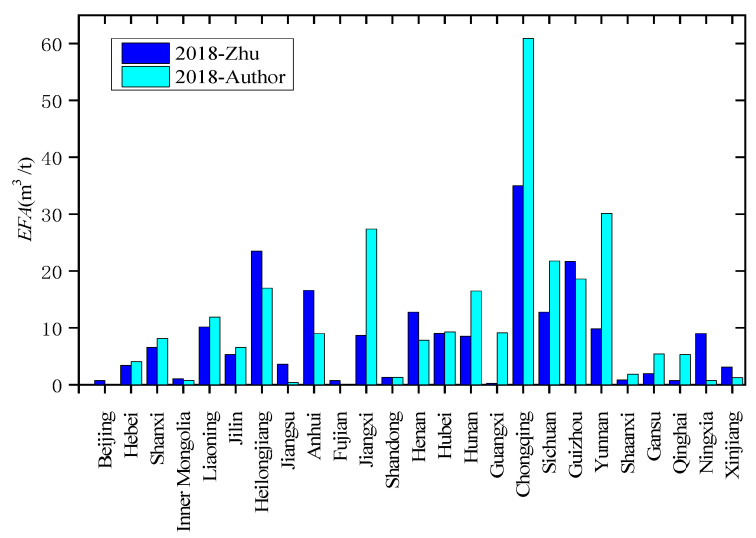
Comparison of CH_4_ emission factors calculated in this paper and those predicted by Zhu et al. [18].

In research on the distribution of gas emission factors in China, Zhu obtained a relatively high degree of recognition for the prediction results based on the coal mine gas data of 787 coal mines collected by SACMS in 2009 [18]. Comparing the CH_4_ emission factors calculated in this paper with the prediction results of Zhu, the calculation results are generally consistent with the model prediction results of Zhu, except in some provinces such as Chongqing, Jiangxi, and Yunnan, as shown in Figure 2. With the policy on coal mine capacity optimization, provinces in southwest China with complex gas geological conditions have almost stopped building new coal mines. Most of the coal mines in Chongqing, Jiangxi, and Yunnan are old mines. In recent years, with the increased mining depth, the gas content of coal seams has also increased significantly. It also shows that the provinces need to further optimize their coal production capacity and increase CH_4_ emission reduction efforts in coal mining industries.

**Figure 3:**
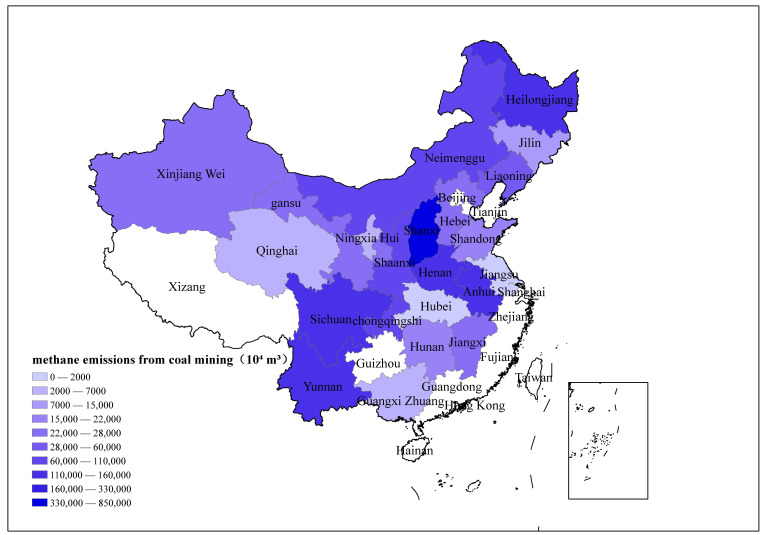
CH_4_ emissions of the coal mining industry in various provinces.

**Figure 4:**
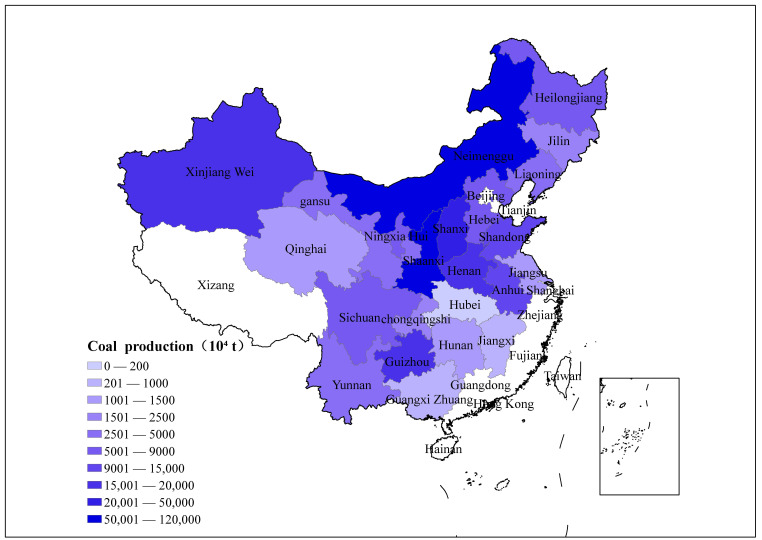
Coal production in various provinces.

According to the China energy statistical yearbook, the coal output and CH_4_ emissions from coal mines are plotted, in 23 major coal producing provinces in China, as shown in Figure 3 and Figure 4, respectively. The results show that without considering utilization, the total CH_4_ emissions of China’s coal mines in 2018 were 23.75 billion m^3^, equivalent to 445.55 Tg carbon dioxide equivalent (co2eq). Among them, Shanxi Province is much higher than other provinces, and its CH_4_ emissions are as high as 8.43 billion m^3^, accounting for 35.5% of the total CH_4_ emissions from the coal mining industry. This is mainly due to the high coal production and high coal mine CH_4_ emission factors in Shanxi. In addition, Guizhou, Heilongjiang, Sichuan, Henan, and Anhui Provinces also have relatively high CH_4_ emissions, which are 3.24 billion m^3^, 1.51 billion m^3^, 1.41 billion m^3^, 1.39 billion m^3^, and 1.21 billion m^3^, respectively. In the future, it is necessary to focus on and vigorously promote the reduction of CH_4_ in coal mines in the provinces, reduce the greenhouse effect caused by the coal production process, and promote the safe and low-carbon development of coal production.

**Figure 5:**
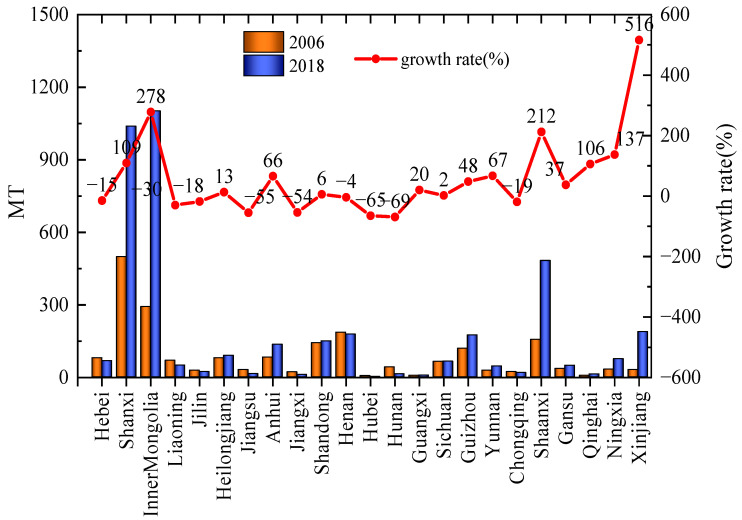
The changes in coal production in China’s major coal-producing provinces.

**Figure 6:**
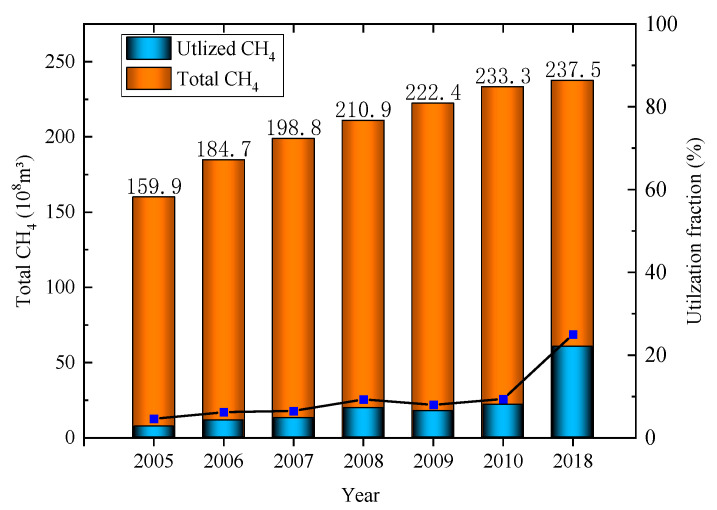
Changes in CH_4_ emissions and CH_4_ utilization from coal mines.

According to the Annual Report on the Development of the Coal Industry [20], China’s underground coal mines’ CH_4_ extraction and utilization amounted to 6.05 billion m^3^ in 2018. Ignoring the methane emissions from open-pit mines, combined with our previous calculation results, it can be estimated that the amount of CH_4_ emitted into the atmosphere during the coal mining process in 2018 was 17.7 billion m^3^, equivalent to 11.8 Tg, and the comprehensive CH_4_ utilization rate was approximately 25%. However, in the report of EDGARv6.0, the predicted value of CH_4_ emissions in 2018 was 20.045 Tg [21], while Liu Gang used national emission factors to estimate methane emissions and drew the conclusion that methane emissions are 18 Tg [22]. Compared with the results of the above studies, the CH_4_ emissions calculated in this paper are quite different. The most important reason is that the pattern of China’s coal industry has been optimized. According to the China Energy Statistical Yearbook, the changes in coal production in China’s major coal-producing provinces between 2006 and 2018 are shown in Figure 5. As can be seen from Figure 5, the focus of coal production is concentrated in Shaanxi, Inner Mongolia, Xinjiang, and other regions with good resource endowments and low gas content in coal mines, and the raw coal production in these regions accounts for 74.3% of the national total. This has also led to significant reductions in coal mine production-weighted CH_4_ emission factors and gas emissions in China. Second, the substantial increase in gas extraction and utilization is also an important reason. The changes in coal mine CH_4_ emissions and CH_4_ utilization in some years are shown in Figure 6 (except for the CH_4_ emission in 2018, which was calculated in this paper, other data are from the China Coal Industry Association released by the China Coal Industry Association. Industry Development Report). Figure 6 shows that with the innovation of technology, the utilization rate of coal mine gas has been significantly improved. Especially after the “Opinions on Further Accelerating the Drainage and Utilization of Coalbed Methane (Coal Mine Gas)” issued by the General Office of the State Council of China in 2013, the utilization of coal mine gas has received more attention, and the utilization level has increased rapidly. The third reason is the closure and elimination of backward coal mines. In 2016, the State Council issued the “Opinions of the State Council on Dissolving Overcapacity in the Coal Industry and Getting Out of the Development Dilemma”. The Opinions require the closure of mines with severe disasters (most of which are high-gas mines), and strictly prohibit the approval of the construction of coal and gas outburst mines with an annual output of less than 0.9 million. The Opinions has resulted in the phasing out of gassy mines, and further reduced the national methane emission factor.

### 3.2. Discussion of the Spatial Differences in CH_4_ Emission Factors

Figure 2 shows that the CH_4_ emission factors in different provinces are significantly different, and those in southwest China are much higher than in others. However, Figure 3 shows that the distribution of CH_4_ emissions is more uniform. This is mainly due to the policy impact of reducing coal production in provinces with high mine gas content. The coal mines in southwest China are deeply exploited and the coal seam methane content is high, so the CH_4_ emission factor in this region is higher than that in other regions. Therefore, although the coal production in the southwest region is small, its CH_4_ emissions are relatively large, among which Sichuan, Chongqing, Guizhou, and Yunnan account for approximately 30% of the national total.

**Figure 7:**
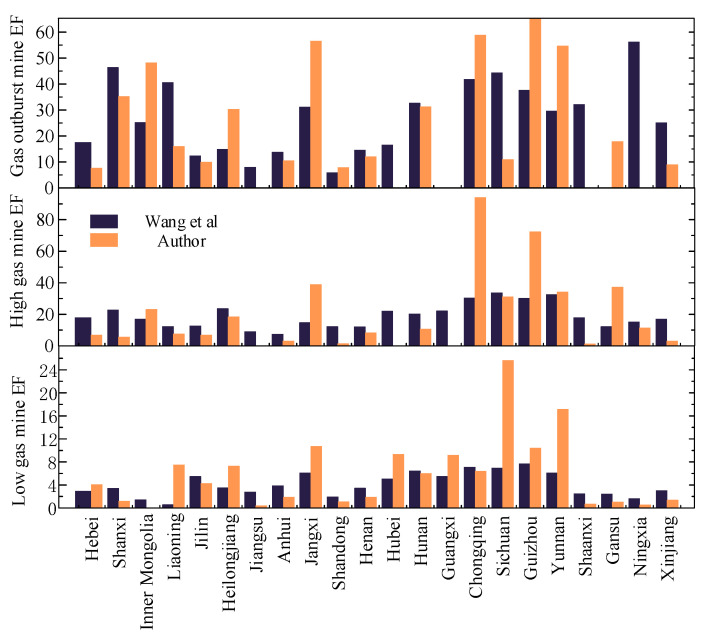
Comparison of CH_4_ emission factors between 2011 [23] and 2018 in this paper.

To further study the spatial distribution of CH_4_ emission factors, this paper classifies and evaluates the coal mines surveyed in each province. These coal mines are divided into three types: low-gas mines, high-gas mines, and outburst coal mines, and the CH_4_ emission factors of the above three types of coal mines in each province are calculated. The comparison between the calculation results in this paper and the calculation results of Wang et al. [23] based on the coal mine data in 2011 is shown in Figure 7. The results show that the emission factors have the same provincial distribution characteristics, although there are differences in individual provinces (e.g., Inner Mongolia, Jiangxi, and Chongqing), which may be due to the optimization of coal mine capacity. The CH_4_ emission factors of high-gas and low-gas mines in southwest China (Sichuan, Chongqing, Guizhou, Yunnan) are significantly higher than those of high- and low-gas mines in north China and northwest China. The calculation results of CH_4_ emission factors of three types of coal mines show that the difference between provincial emission factors is larger than that of national emission factors. The coal mine CH_4_ emission factor of low-gas mines is the smallest at 0.01 m^3^/t, and the highest in Yunnan Province, reaching 17.14 m^3^/t. Among the high-gas mines, the emission coefficient of coal mines in Shaanxi is the lowest at 1.3 m^3^/t, and Chongqing has the highest emission factor of 94 m^3^/t. Therefore, China should focus on CH_4_ emissions in the southwest and increase CH_4_ emission reduction efforts in this region.

## 4. Recovery and Utilization Technology of Coal Mine CH_4_

China’s energy endowment is characterized by abundant coal resources and a lack of oil and natural gas. Therefore, coal will still be the main energy source for China for a long time in the future. The challenges cannot be ignored, regarding the CH_4_ emissions in the coal industry, and the fundamental way to solve them is to improve the level of recovery and utilization of CH_4_ in coal mines. Compared with countries such as the United States and Australia that have successfully developed commercial coalbed methane mining, China’s coalbed methane industry is still in its infancy. In recent years, China has continuously strengthened policy guidance on the development and utilization of coalbed methane. In 2021, the State Council of China issued the “Working Guidance for Carbon Dioxide Peaking and Carbon Neutrality in Full and Faithful Implementation of the New Development Philosophy” and the “Action Plan for Carbon Dioxide Peaking Before 2030“. Both plans mentioned the need to accelerate the large-scale development of unconventional oil and gas resources such as coalbed methane [24].

The occurrence conditions of CBM resources in China are complex, and the development technology of coalbed methane resources is heavily needed, but the regional adaptability of the existing technology is poor. Many technologies that have been found to be feasible by countries such as the United States and Australia are not applicable in China, so it is necessary to explore CBM development technologies suitable for China’s geological conditions. For example, as 82% of the soft and low permeability coal seams in China’s coal mining areas, the research and application of horizontal well staged fracturing, and extraction technology in the roof strata of broken soft and low permeability coal seams have achieved good application results in some mines. The technology can not only greatly improve the extraction rate, but also reduce the gas concentration in the return air tunnel of the mine face by 30% to 50%, improving the safety level of coal mines [25].

The technologies and equipment for reducing methane emissions of coal mines mainly include gas drainage and low-concentration gas utilization. With more advanced drilling equipment, coal mines can construct gas drainage holes more conveniently and economically to increase the CH_4_ drainage volume. For poor gas permeability in coal seams that are difficult to extract gas, methods that make it easier to extract can be used, such as hydraulic fracturing, deep hole blasting pre-fracture, and hydraulic cutting slits, to increase the porosity of the coal seam [26,27]. In terms of gas utilization, the most critical is gas purification technology and low-concentration gas power generation technology. Greater development of these technologies and their widespread use by coal mining companies can help reduce methane emissions.

**Figure 8:**
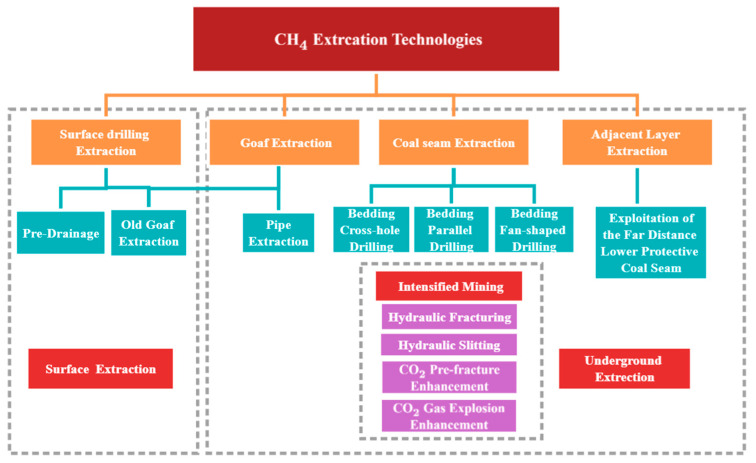
Coal mine CH_4_ extraction technologies with promotion potential.

Through field investigations in Shanxi, Henan, Guizhou, Sichuan, and other locations, this paper summarizes the CBM development technologies with better application effects, as shown in Figure 8. At present, the relatively mature CBM development model includes a variety of CBM development technologies, including surface hole extraction and underground borehole extraction. The surface hole extraction includes pre-pumping before ground drilling and the extraction of the old goaf by ground grilling; the underground borehole extraction can be divided into the extraction of the coal seam, the extraction of the adjacent layer, and the extraction of the goaf. The coal seam drainage can be divided into cross borehole extraction along with the layer, extraction of parallel borehole along with the layer, and extraction of sector boreholes along with the layer according to its drilling arrangement. In addition, a variety of enhanced extraction technologies can be used to improve the CBM extraction rate of coal seam boreholes, such as hydraulic fracturing, hydraulic slotting, CO_2_ presplit blasting, and permeability enhancement by CO_2_ explosion.

**Figure 9:**
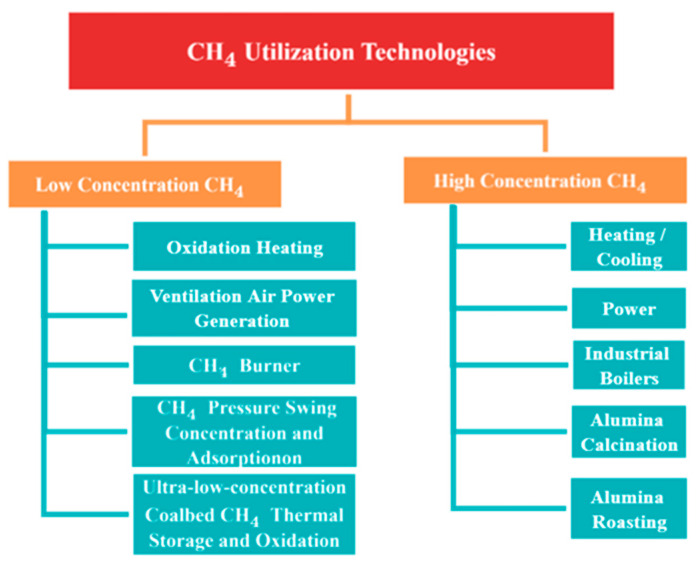
CBM utilization technologies with good technical prospects.

Another limitation is the utilization of CBM technology. Theoretically, mines could be completely decarbonized. However, this does not apply to volatile methane. All that can be done to reduce emissions is to fully electrify mines using renewable energy sources. According to the “2018 Annual Report on Coal Industry Development”, the utilization rate of gas extracted from coal mines in China is only 44.8%. This is mainly restricted by technology, especially because the low-concentration gas utilization technology is still immature and the economic benefit is poor, so it is difficult to realize the large-scale utilization of coal mine CH_4_. Therefore, the development of coalbed methane utilization technology is very important. Based on this investigation, we summarize the CBM utilization technologies with good technical prospects, as shown in Figure 9.

High-concentration coalbed methane can be used as gas for residents and offices, as well as for alumina roasting, coalbed methane power generation, and industrial boilers. Among them, mine gas power generation technology is the most promising method for large-scale applications. The gas extraction equipment transports the underground gas to the ground gas power station to generate electricity. This technology is suitable for coal mines where the pure gas drainage volume of the gas drainage system is approximately 1 million m^3^/year and the gas concentration is between 6% and 25%.

The utilization technology of low-concentration coalbed methane is very complicated. The low-concentration coalbed methane pressure swing concentration and adsorption technology can enrich the feed gas with a concentration of more than 15% to 95% through two-stage upgrading at 150 kPa [28]. The technology of low-concentration coalbed methane oxidation exothermic heating wellbore is to mix air into 1.2% low-concentration coalbed methane, and then oxidize at high temperature to release heat to heat wellbore fresh air. Coal mine ventilation air power generation technology mixes the CH_4_ with a concentration of less than 8% from coal seam extraction and the coal mine ventilation air (very low CH_4_ concentration) to make the mixed gas concentration reach approximately 1%, and then uses it for power generation. The use of low-concentration coalbed methane burner technology and equipment can make the safe combustion of low-concentration CH_4_ reach 500 m^3^/h, and the CH_4_ combustion rate can reach 100%. Using low-concentration coalbed methane concentration technology and equipment, through three times the concentration, the methane concentration can be enriched from 30% to more than 90%. Ultralow-concentration coalbed methane thermal storage and oxidation technology are based on low-concentration coalbed CH_4_ transportation technology, intelligent mixing technology of various concentrations of CH_4_ technology, and low-concentration CH_4_ thermal storage and oxidation equipment, which solves the issue of large gas concentration fluctuations, and its CH_4_ oxidation rate can reach more than 98%.

## 5. Conclusions and Recommendations

### 5.1. Conclusions

This paper adopts the regional CH_4_ emission accounting method proposed by the IPCC, combines the survey data of nearly 1840 coal mines to establish the CH_4_ emission inventory of China’s coal mining industry in 2018, and analyzes the distribution characteristics of CH_4_ emissions of coal mines in different provinces. The main conclusions obtained are as follows:

(1) The total CH_4_ emissions of China’s coal mines in 2018 were 23.75 billion m^3^. After subtracting recovered and utilized CH_4_, 17.7 billion m^3^ of CH_4_ was emitted into the atmosphere. The CH_4_ emissions of different provinces vary greatly. Among them, Shanxi Province is much higher than other provinces, and its CH_4_ emissions are as high as 8.43 billion m^3^, accounting for 35.5% of the total CH_4_ emissions. In addition, Guizhou, Heilongjiang, Sichuan, Henan, and Anhui Provinces also have relatively high CH_4_ emissions which are 3.24 billion m^3^, 1.51 billion m^3^, 1.41 billion m^3^, 1.39 billion m^3^, and 1.21 billion m^3^, respectively.
(2) The CH_4_ emission factor of China’s coal mining industry in 2018 is 6.77 m^3^/t, which is lower than the predicted value of the IPCC and other institutions and scholars. The most critical reason for this lower value is that China’s coal production centers are moving to areas with low gas content. There are significant differences among different provinces. Chongqing has the highest CH_4_ emission factor 60.9 m^3^/t. The CH_4_ emission factors of Inner Mongolia, Shaanxi, and Xinjiang in the northwestern region are relatively low, not exceeding 2 m^3^/t.
(3) CH_4_ emission factors for coal mining are related to the geological conditions of different regions. Coal mines in southwest China have the highest CH_4_ emission factors, and although coal production in this region is small, the total amount of CH_4_ emissions from coal mining is large.
(4) The growth of CH_4_ emissions from coal mines in China is slowing, and the CH_4_ utilization rate is gradually increasing. This is due to the policy of China’s coal industry to eliminate outdated production capacity, that is, to close most high-gas mines, increase the production of coal mines with low gas and high production, and improve CH_4_ utilization technology.

### 5.2. Recommendations for CH_4_ Emission Reduction in Coal Mines

Optimization of coal mining technology. In the field of coal mining, it is necessary to continuously increase the exploration and development of coalbed methane and reduce methane emissions by means of policy guidance and the use of market mechanisms. In addition, it is also necessary to vigorously promote the application of intelligent extraction and other technologies, break through the technical bottlenecks such as soft coal seam hole collapse and waste coal mine gas development, improve the gas extraction rate, and reduce the CH_4_ emissions of coal mines from the source.

Expand the CH_4_ utilization method and improve the comprehensive utilization rate of CH_4_. The most urgent task is to increase key technical and economically feasible technological breakthroughs in coal mine CH_4_ utilization, reduce utilization costs, and provide technical support and guarantees for coal mine CH_4_ emission reduction. For coal mine CH_4_ of different concentrations, it is necessary to actively carry out diversified and comprehensive utilization of coal mine gas such as civil gas, industrial boilers, coal mine CH_4_ power generation, CH_4_ purification and utilization, and oxidation heating, in combination with various technical conditions of coal mine CH_4_ utilization.

It is also necessary to enrich CH_4_ measurement methods and improve CH_4_ measurement accuracy. CH_4_ emissions from mines have been variable due to changes in coal mining and gas release rates. The choice of sensor measurement location and data collection frequency can also lead to uncertainty in the measurement results. As a result, this direct “bottom-up” approach tends to underestimate CH_4_ emissions. To obtain comprehensive and complete emission data, it is usually necessary to combine the “top-down” method. By means of vehicle-mounted monitors, aircraft, satellites, drones, and tower networks, it is more convenient to obtain the total data of large spatial scales and all emission sources and to facilitate the calibration of microscopic data.

In addition, methane emissions can be reduced by improving the efficiency of fuel combustion. By improving energy efficiency, energy demand can be reduced, so as to reduce the exploitation of coal and oil and gas, and achieve the goal of CH_4_ emission reduction.

## Figures and Tables

**Table 1:** Methane emission factors for coal mining [13].

Types of Coal Mines		Method 1		Method 2	
		Underground Mine (m^3^/t)	Open-Pit Mining (m^3^/t)	Underground Mine (m^3^/t)	Open-Pit Mining (m^3^/t)
mines activities	high gas	25	2.0	country or region measurements	
	medium gas	18	1.2		
	low gas	10	0.3		
post-mining	high gas	4.0	0.2	10–30% of the gas content in coal; usually 0.1	

**Table 2:** Survey and calculation results of coal mining and CH_4_ emission in Qinghai Province.

Mine Name	Mining Depth	Actual Production (10^4^ tons)	*ea* (m^3^/min)	*EA* (10^4^ m^3^)	*EFA* (m^3^/t)
No.3 Mine of Mule	500	14.4	3.5	183.96	12.78
Tiemai Mine	283	7.9	1.2	63.07	7.98
Chaidaer Mine	182	39.8	12.5	657.00	16.51
Chaidal Pioneer Mine	300	12.9	13.4	704.30	54.60
No.1 Mine of Yuka	570	273	14.90	782.99	2.87
Dameigou Mine	300	110.8	1.17	61.50	0.56
No. 1 Mine of Datouyang	288	4	0.63	33.32	8.33
No. 2 Mine of Datouyang	480	13	0.63	33.32	2.56
Lvcaogou Coal Mine	300	8.1	0.77	40.47	5.00
total (or average)	—	483.9	48.71	2559.93	—

## Abbreviations

CMM	Coal Mine Methane
IPCC	Intergovernmental Panel on Climate Change
GWP	Global Warming Potential
IEA	International Energy Agency
CSY	China Statistical Yearbook
CESY	China Energy Statistical Yearbook
USEIA	United States Energy Information Administration
SACMS	State Administration of Coal Mine Safety Supervision of China
CBM	Coal Bed Methane
